# Review of Newly Identified Functions Associated With the Heat-Labile Toxin of Enterotoxigenic *Escherichia coli*

**DOI:** 10.3389/fcimb.2019.00292

**Published:** 2019-08-13

**Authors:** Qiangde Duan, Pengpeng Xia, Rahul Nandre, Weiping Zhang, Guoqiang Zhu

**Affiliations:** ^1^College of Veterinary Medicine, Institute of Comparative Medicine, Yangzhou University, Yangzhou, China; ^2^Joint International Research Laboratory of Agriculture and Agri-Product Safety of Ministry of Education of China, Jiangsu Co-innovation Center for Prevention and Control of Important Animal Infectious Diseases and Zoonoses, Yangzhou, China; ^3^Department of Microbiology and Immunology, Uniformed Services University, Bethesda, MD, United States; ^4^Department of Pathobiology, University of Illinois at Urbana-Champaign, Urbana, IL, United States

**Keywords:** LT (heat-labile toxin), ETEC, enterotoxicity, adherence, adjuvanticity

## Abstract

Heat-labile toxin (LT) is a well-characterized powerful enterotoxin produced by enterotoxigenic *Escherichia coli* (ETEC). This toxin is known to contribute to diarrhea in young children in developing countries, international travelers, as well as many different species of young animals. Interestingly, it has also been revealed that LT is involved in other activities in addition to its role in enterotoxicity. Recent studies have indicated that LT toxin enhances enteric pathogen adherence and subsequent intestinal colonization. LT has also been shown to act as a powerful adjuvant capable of upregulating vaccine antigenicity; it also serves as a protein or antigenic peptide display platform for new vaccine development, and can be used as a naturally derived cell targeting and protein delivery tool. This review summarizes the epidemiology, secretion, delivery, and mechanisms of action of LT, while also highlighting new functions revealed by recent studies.

## Introduction

Enterotoxigenic *Escherichia coli* (ETEC) is a type of *E. coli* that is characterized by its ability to produce heat-labile (LT) and/or heat-stable (ST) enterotoxins. ETEC is a leading bacterial cause of diarrhea in children younger than 5 years in developing countries, international travelers, and also neonatal and post-weaning animals (Nagy and Fekete, 2005; World Health Organization, 2006; Crossman et al., 2010; Kotloff et al., 2013; Lamberti et al., 2014; Platts-Mills et al., 2015). ETEC-induced diarrhea remains a serious problem with an estimated 840 million infections and over 3,800,000 deaths worldwide each year. LT is one of the main enterotoxins produced by ETEC (Gupta et al., 2008). LT is categorized as either type I LT (referred to hereafter as LT) or type II LT (LTIIa, b, c) depending on its antigenic capacity and associated genetic sequence (Hajishengallis and Connell, 2013). The amino acid sequences of the LTA subunit of the LT and LTII enterotoxins are highly homologous, whereas, the amino acid sequences of the LTB subunit are highly divergent between LT and LTII (Pickett et al., 1986; Connell, 2007). The A subunit of LT-IIc share 79 and 72% amino acid sequence homology with the A subunit of LT-IIa and LT-IIb, respectively. However, the B subunit of LT-IIc exhibited only 53% amino acid sequence similarity to the B subunit of LT-IIa and only 54% amino acid sequence similarity to the B subunit of LT-IIb (Nawar et al., 2010). In addition, LT-IIc exhibits potent immunomodulatory properties that are different from those induced by LT-IIa or LT-IIb (Nawar et al., 2011). While LT-IIa and LT-IIb appear to drive a more balanced Th1/Th2 immune response to a co-administered antigen; LT-IIa or LT-IIb, LT-IIc has a greater capacity to drive antigen-specific Th1 type immune responses. Table 1 summarizes differences at the organism, source, and amino acid identity among LT variants, and Table 2 showed their differences at properties. LT can be further categorized into LTh derived from humans and LTp derived from piglets. LT II is predominantly associated with ETEC isolated from animals, while the LT gene is highly prevalent in ETEC strains isolated from both humans and animals. The LT gene was detected in 57.7% of ETEC isolates associated with porcine post-weaning diarrhea (PWD) in the US (Zhang et al., 2007). In a separate study, it was revealed that approximately 60% of field ETEC isolates associated with human diarrhea expressed either LT alone (27%) or LT with ST (33%) (Isidean et al., 2011). Given the global distribution of LT-specific ETEC strains and the fact that no effective vaccines are currently available, it is likely that infections caused by these pathogens will have a large impact on global public health.

**Table 1:** Organism, source, and similarity index of variants of LT.

**Variants of LT**	**Organism**	**Source**	**Similarity index (%)**	
			**LTA subunit**	**LTB subunit**
LTh-I^a^	ETEC H10407	Human	100	100
LTp-I	UMNK88	Pig	98.8	96.1
LT-IIa	SA53	Water buffalo	52.7	16.3
LT-IIb	EC41	Cooked beef	56.4	17.1
LT-IIc	OS-1	Poultry	55.9	20.7

a *The amino acids of LTh was used as the reference when the similarity index was done*.

**Table 2:** Distinct properties of LT and LT-II.

	**LTA** **(amino acid)**	**LTB** **(amino acid)**	**Cytotoxicity**	**Receptor(s)**	**Th1 or Th2 response**	**References**
LTh	240	103	++++	High: GM1 Weaker: GM2, GD1b, LPS, A, B-type blood sugars	Th2	Fukuta et al., 1988; Horstman and Kuehn, 2002; Holmner et al., 2007; Mudrak and Kuehn, 2010
LTp	240	103	++++	High: GM1 Weaker: GM2, GD1b, LPS, A, B-type blood sugars	Th2	
LT-IIa	241	100	+++	High: GD1b Weaker: GD1a, GM1, GM2, GM3, GT1b, GQ1b, GD2	Th1 = Th2	Fukuta et al., 1988; Hajishengallis and Connell, 2013
LT-IIb	243	99	++	High: GD1a Weaker: GT1b, GM1b, GD1α, GM2, GM3	Th1 = Th2	Fukuta et al., 1988; Berenson et al., 2010; Hajishengallis and Connell, 2013
LT-IIc	241	98	+	High: GD1a, GM1, GM2, GM3, GD1α Weaker: GQ1b	Th1 > Th2	Nawar et al., 2010, 2011

LT holotoxin is initially assembled in the periplasm and then secreted to the outer membrane by one of the following two systems: (1) via the classic type II secretion system (T2SS), (2) through outer membrane vesicles (OMVs) released by ETEC. Even in the presence of the T2SS, a majority of secreted LT remain associated with OMVs because its B subunits bind lipopolysaccharide (LPS) (Horstman and Kuehn, 2000, 2002). It has been previously been reported that the pentameric LTB subunit structure is required for the effective secretion of the LT holotoxin. It has been suggested that the N-terminal alpha1 helix of LTB is not only required to maintain the structural stability of LT, but is also required for effective binding to GM1 receptors (Alone and Garg, 2008). Recently, Heggelund et al. used surface plasmon resonance spectroscopy to reveal that amino acid residues 7, 18, 94, and 95 of the LTB subunit most dramatically affect its binding affinity and specificity (Heggelund et al., 2019). Deletion of the LTB subunit protein affects the secondary structure, impairs its secretion and reduces GM1 receptor affinity. Several LTB subunit mutants were shown to impair the secretion efficiency of LT. The levels of LT secretion are reduced by half among some LTB mutants (Q3K, E11K, and L25E), due to impaired LT secretion (Mudrak et al., 2009; Mudrak and Kuehn, 2010). In addition to the classical type II secretion system, LT can also be secreted through OMVs released by ETEC (Horstman and Kuehn, 2000). It was reported that a vast majority of secreted LT remain attached to the LPS in OMVs on the surface of *E. coli* (Horstman and Kuehn, 2002). Thus, the delivery of LT is predominantly facilitated by the LT-bound OMVs (internal and external) form, with the 3rd, 11, 46, and 47 residues played an important role in LTB binding to LPS (Mudrak et al., 2009). However, a different study concluded that neither LT nor CT binds to the surface of *Vibrio* cells, and the disparity may be caused by structure differences in the LPS produced by ETEC and *Vibrio* (Horstman et al., 2004). The interactions between LT and OMVs as a delivery pathway facilitate an intimate contact between vesicles and the host cells, leading to intoxication. A recent study found that EatA, a serine protease autotransporter of the Enterobacteriaceae (SPATE) protein, can reduce bacterial adhesion and accelerate delivery of the LT by degrading an adhesin called EtpA (Roy et al., 2011). The effective ETEC-host interaction is an essential prerequisite for LT toxin delivery by ETEC. Further, the delivery of LT toxin will enhance ETEC adherence and may increase the receptors of ETEC virulence factors expression. It is important to note that (1) motility, (2) host cell contact (Dorsey et al., 2006), and (3) adhesion (Roy et al., 2012) are each absolute requirements for effective toxin delivery. However, the precise mechanisms that underlie the secretion and delivery of LT remain to be fully elucidated.

**Figure 1:**
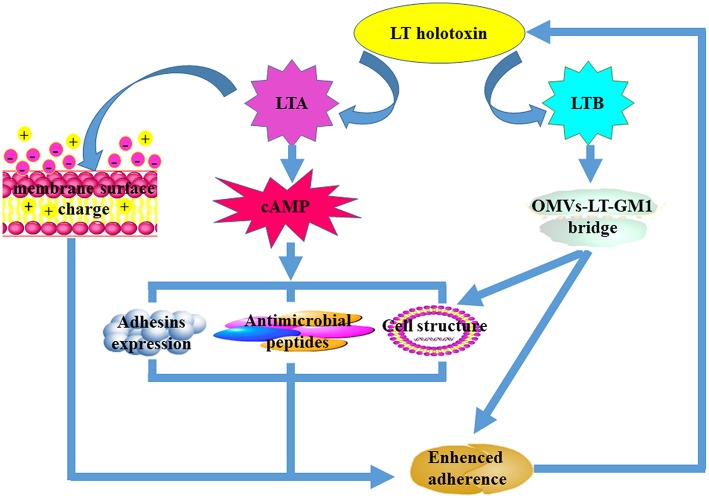
The mechanisms that underpin LT-mediated enhancement of bacterial adherence. Both LTA and LTB subunits are required for LT-mediated enhancement of bacterial adherence. The LTA subunit improves bacterial adherence predominantly by decreasing the cell membrane surface charge and the expression of antimicrobial peptides, increasing the expression of adhesins on the surface, and altering host cell structure. The LTB subunit facilitates bacterial adherence mainly by forming the “OMVs-LT-GM1” bridge after interaction with the GM1 receptors. Alteration of the host cell membrane can also enhance bacterial adherence.

LT enterotoxicity has been extensively investigated and is well characterized as shown in Figure 1 (Harford et al., 1989; Tsuji et al., 1990). LT is one of the A_1_B_5_ toxin family proteins, and is similar in structure, function and pathogenesis to cholera toxin (CT) secreted by *Vibrio cholerae* (*V. cholerae*). Briefly, the LTB subunit binds to GM1 receptors with high affinity at the host mucosa surface and enters into epithelial cells via endocytosis. In addition to GM1, LT is capable of binding both blood group A and B determinants by interacting with a novel carbohydrate binding site located at the top of the B-subunit interfaces, distinct from the GM1 binding site (Angström et al., 2000). Following internalization into the endoplasmic reticulum (ER), the LTA peptide is cleaved into A1 and A2 fragments. The ADP-ribosylation activity of the LTA1 fragment activates adenylyl cyclase (AC) thereby elevating intracellular cAMP levels (Gill and Richardson, 1980; Field et al., 1989). Elevated cAMP levels result in the activation of protein kinase A (PKA)-dependent pathways, which inhibits Na^+^ absorption through sodium-hydrogen (Na^+^/H^+^) exchangers (NHE2 and NHE3) and stimulates Cl^−^ secretion by phosphorylation of the cystic fibrosis transmembrane regulator (CFTR) (Field et al., 1989; Viswanathan et al., 2009). The altered electrolyte and water balance finally lead to severe volumes of watery diarrhea.

## LT Improves Bacterial Adherence and Intestinal Colonization

LT contributes to ETEC pathogenesis by facilitating the initial adherence and subsequent intestinal colonization of the murine and porcine intestinal mucosa with the subsequent production of watery diarrhea (Berberov et al., 2004; Zhang et al., 2006). The phenomenon of LT-mediated enhancement of bacterial adherence was first observed in a gnotobiotic piglet model of a F4+ ETEC strain infection (Berberov et al., 2004). In this model, a F4+ ETEC mutant unable to produce LT exhibited a dramatic reduction in the colonization of the jejunum and ileum when compared to the wild-type or complementation strain. Likewise, in a separate study, it was reported that expression of F4ac fimbriae, which enable adhesion to small intestine epithelial cells, was significantly increased in a nonpathogenic *E. coli* field isolate when LT was genetically introduced into the strain (Zhang et al., 2006). Furthermore, similar to the results of a study investigating porcine ETEC infection of gnotobiotic piglets, the former study revealed that elaboration of LT can promote early colonization of human ETEC in a murine model. Subsequently, it was reported that endogenously produced or exogenously added LT substantially enhances the adherence of K88+ fimbriated ETEC to the cultured piglet epithelial cell line, IPEC-J2 (Johnson et al., 2009). Moreover, expression of LT holotoxin preferentially enhances G58-1 strain adherence compared with expression of the LTB subunit alone; mutants lacking ADP-ribosylation enzymatic activity facilitated the same levels of adherence as cells transformed with the LTB subunit construct (Santiago-Mateo et al., 2012; Fekete et al., 2013). In addition, to facilitate ETEC adherence, a recent study revealed that the presence of LT in the gut also significantly promotes *Salmonella enterica* colonization of pig intestinal cells (Verbrugghe et al., 2015). Up until now, the effect of LT on the enhancement of bacterial adherence has been predominantly studied in enteric pathogens, particularly ETEC strains. However, it is still unclear whether LT also facilitates the other species adherence to host cells. Interestingly, LT has a stronger effect on adherence in F4+ ETEC compared with ETEC carrying other fimbriae.

Although, the intestinal mucus layer forms the first line of host defense against pathogenic microorganisms, it has been reported that LT enhances bacterial adherence by mechanically disrupting the intestinal mucus layer and altering the function of intestinal cells. In addition, LT alters the structure and composition of the intestinal epithelial mucin layer by reducing mucin 4 expression in goblet cells; this phenomenon subsequently leads to bacterial adherence (Verbrugghe et al., 2015). Another study demonstrated that LT can enhance mucin-2 expression and EtpA-dependent ETEC adhesion *in vitro*. However, it is also reported that the significant numbers of bacteria can adhere to small intestine epithelial cells in MUC2^−/−^ mice than C57BK/6 mice *in vivo* (Kumar et al., 2016). These results indicated a complex relationship between bacterial adhesion, colonization and LT expression. In addition, LT-mediated inhibition of ascorbic acid uptake (Subramenium et al., 2019) and reduction to the surface charge of the host plasma membrane (Fekete et al., 2013) both affect the function of intestinal cells and promote non-specific adherence. These phenomena cause interference with the host innate immune response to infection. Human antimicrobial peptides (AMPs) elicit antimicrobial activity against a broad range of enteric pathogens and play critical roles in host innate immune responses. LT was shown to repress the expression of Cathelcidin (LL-37) and B-defensin 1 (HBD-1), thereby facilitating bacterial adherence and subsequent colonization (Chakraborty et al., 2008). In addition to AMPs, LT can also inhibit the expression of pro-inflammatory cytokines including tumor necrosis factor α (TNF-α) and interleukin 8 (IL-8) both of which play important roles in the host innate immune response (Turcanu et al., 2002; Glenn et al., 2009). Finally, LT also enhances adherence by forming an “OMV-LT-GM1” bridge and influencing the expression of other virulence determinants associated with the pathogens. These results indicated that LT-mediated bacterial adherence is mainly attributed to: (1) alteration the structure of intestinal cells; (2) repression of host innate immune responses like inhibiting AMPs and proinflammatory cytokines production; (3) enhancement of the expression of host receptors for adhesins; (4) increase in expression of virulence factors. However, the precise mechanisms that underpin LT-mediated bacterial adherence remain to be elucidated.

It is still unclear which subunit plays a central role in enhancing LT-mediated bacterial adherence. Some studies have suggested that bacterial adherence is enhanced by LT because LT elevates cAMP levels through the ADP-ribosylation activity of the LTA subunit. These studies report that the enhancement of bacterial adherence by LT subverts innate immune responses by blocking host NF-κB activation (Wang and Hardwidge, 2012) and activating the MAPK pathway using cAMP-dependent mechanisms (Johnson et al., 2009; Wang et al., 2012). Given that cAMP is an important secondary messenger in many essential host-signaling pathways, increases in the levels of adherence facilitated by LT appear primarily to be due to increases in the levels of cAMP in host epithelial cells. However, Fekete et al. (2013) reported that both the enzymatic LTA subunit and the adhesive LTB subunit are responsible for LT-enhanced K88+ ETEC adherence to IPEC-J2 cells. In contrast to previous reports, Fekete et al. (2013) suggest that the improved adherence is primarily due to the bridge mediated between the LTB subunit and specific GM1 receptors.

**Figure 2:**
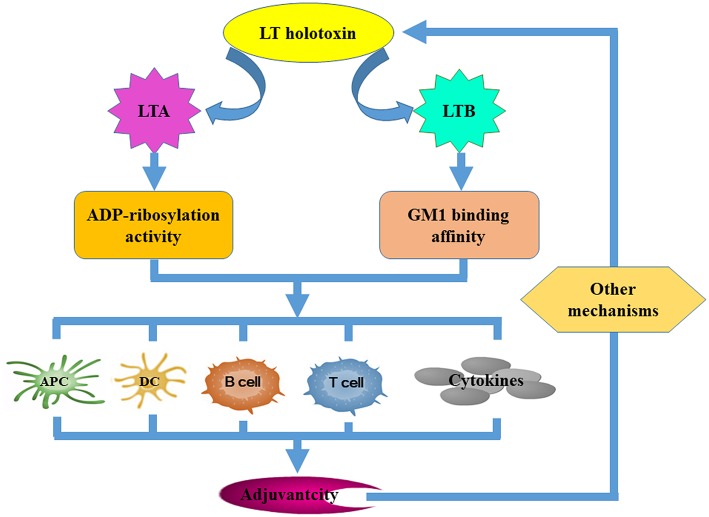
The mechanisms that underpin LT derivative adjuvant activity. Several LT-specific activities facilitate the improvement of vaccine efficacy. Both the ADP-ribosylation activity of the LTA subunit and the GM-binding affinity of the LTB subunit are required for LT to exert its adjuvanticity. Both of these activities contribute to adjuvanticity by improving the antigen-presenting capacity of APCs, promoting DC maturation and activation, activating B and T cells, and inducing cytokine secretion. Other mechanisms independent of the ADP-ribosylation activity of LTA and the GM1-binding affinity of LTB may be required.

It is now clear that both subunits contribute to LT-enhanced bacterial adherence (Figure 2), even though it remains unclear which subunit plays the central role. LTA contributes to LT-enhanced adherence and is mainly dependent on its ADP-ribosylation activity to facilitate increases in cAMP levels. An elevation in cAMP levels may regulate expression of other bacterial virulence factors and host immune responses. For example, down-regulation of AMPs in the intestine and alterations to the host epithelial cell morphological structure facilitate bacterial adherence to host cells (Yu et al., 2012). An alteration to the surface charge of the host plasma membrane can also increase the levels of non-specific contact between bacteria and host cells. It is likely that the LTB subunit contributes to LT-enhanced adherence by initially forming an “OMV-LT-GM1” bridge to enhance specific binding between bacteria and host cells. After this, it is likely that LTB facilitates LTA subunit internalization into host epithelia after binding to GM1 receptors which are abundant in the lipid rafts of host cells.

## LT as an Adjuvant and Associated Mechanisms of Action

Although the holotoxin-structured LT molecule is a strong adjuvant, enterotoxicity prevents native LT from being applied clinically. In an attempt to generate nontoxic derivatives of LT that retain significant adjuvanticity, there has been a major focus on reducing or abolishing its ADP-ribosylation activity by substituting the residues of the LTA subunit. The mutant forms of LT that have been generated thus far include a monomeric mutant LT (mLT), a double mutant LT (dmLT), and a triple mutant LT (tmLT) (Okamoto et al., 1988; Tsuji et al., 1990; Douce et al., 1995; Di Tommaso et al., 1996; Giannelli et al., 1997; Kato et al., 1997; Giuliani et al., 1998; Norton et al., 2011; Zhang et al., 2013). The LTS61K mutant was the first mutant generated that was capable of diminishing the toxicity of LT while maintaining the adjuvant properties of the toxin (Harford et al., 1989). Several mLT mutations were also generated; the latter mutants (including R7K, E112K, V53E/D, S63K, A72R, V97K, Y104K/D, S114K/E, R192G, and L211A) focused on reducing the ADP-ribosylation activity of LT (Tsuji et al., 1990; Pizza et al., 1994; Chong et al., 1998; Norton et al., 2011). The prototype of mLT is LTR192G, where a glycine at position 192 was substituted for an arginine. Position 192 is a proteolytically sensitive site in the A-subunit that is essential for separating the A1 and A2 segments. In both *in vitro* assays and animal studies, mLT shows reduced toxicity and has similar adjuvant activity as the native LT. However, 16.7% of volunteers develop mild to moderate diarrhea when orally administrated 100 μg of LTR192G (Clements and Norton, 2018). In order to further attenuate the enterotoxicity of the mLT mutants, a novel dmLT (LTR192G/L211A) was created by substitution of an alanine for a leucine at amino acid 211 (L211A) of mLT (LTR192G) (Norton et al., 2011). The dmLT mutant resulted in 1,000 times less cAMP in cultured epithelial cells and no detectible fluid accumulation while maintaining immunogenicity and adjuvanticity when compared to the native LT toxin in the patent mouse assay. Research carried out up until now suggests that dmLT is a powerful adjuvant that enhances antigen-specific systemic and mucosal immune responses by parenteral or mucosal immunization (Coleman et al., 2012; Kim et al., 2012; Lee et al., 2014; Norton et al., 2015; Frederick et al., 2018). In our laboratory, we generated a triple mutant LT toxoid [LT (S63K/R192G/L211A), tmLT]; this was fused with three copies of STa toxoid (STaA14Q) to generate the 3 × STa (A14Q)-tmLT fusion protein (Zhang et al., 2013). The fusion antigen eliminates the capacity to induce cAMP in T84 cells and showed no adverse effects in immunized mice. These results indicate that dmLT or tmLT may represent effective and attractive adjuvants for future vaccines. The characteristics of types of LT mutants used as adjuvants for vaccine were shown in Table 3.

**Table 3:** The characteristic of types of LT mutants as adjuvant.

**Types of LT mutants**	**Toxicity**	**Adjuvanticity**	**Routes of administration**	**References**
mLT	Very low or completely defective	Strong	Parenteral and mucosal	Ryan et al., 2000; Barrette et al., 2011; Byrd and Boedeker, 2013
dmLT	Completely defective	Strong	Parenteral and mucosal	Norton et al., 2011, 2015; Lalsiamthara et al., 2016; Duan et al., 2017
tmLT	Completely defective	Strong	Parenteral and mucosal	Zhang et al., 2013

The nontoxic LT or LTB subunits have been used as adjuvants when administrated with a range of antigens (Sjökvist Ottsjö et al., 2013; Nandre and Lee, 2014; Qi et al., 2015) by various routes of administration (Fingerut et al., 2006; Song et al., 2008; Zhang et al., 2009; Nandre et al., 2016; Duan and Zhang, 2017). It has been reported that LT can enhance the immunogenicity of antigens from bacteria, viruses, and fungi thereby improving the efficacy of vaccines (Weltzin et al., 2000; Bonenfant et al., 2001; Romero et al., 2009; Sun et al., 2013; Thiam et al., 2015). The LTS63K and LTR192G mutants are widely used as adjuvants for various candidate vaccines. LTS63K has been demonstrated to act as a potent adjuvant when administrated with Keyhole limpet hemocyanin (KLH) of *Bordetella pertussis*, foot-and-mouth disease virus peptide and colonization factor antigen I (CFA/I) fimbriae of ETEC by the mucosal or parenteral routes (Ryan et al., 2000; Barrette et al., 2011; Byrd and Boedeker, 2013). In our previous study, we demonstrated that when three copies of the STa toxoid mutant were fused to LTR192G, both neutralized anti-STa and anti-LT antibodies were induced in mice that had been immunized with this fusion protein (Zhang et al., 2010; Liu et al., 2011, 2015). dmLT was first developed by Norton and used as an oral adjuvant for tetanus toxoid. Furthermore, dmLT has been shown to be an effective mucosal adjuvant for *Helicobacter pylori* (*H. pylori*), IpadB/IpadD of *Shigella* and poliovirus vaccines (Sjökvist Ottsjö et al., 2013; Lee et al., 2014; White et al., 2014; Norton et al., 2015). Although dmLT was first used as a mucosal adjuvant, studies have also revealed the mutant as an effective parenteral adjuvant (Lalsiamthara et al., 2016). It has previously been shown that *Salmonella enteritidis* live-attenuated vaccines with dmLT as an adjuvant induce robust immune responses (Lalsiamthara et al., 2016). We also demonstrated that ETEC adhesin multi-epitope fusion antigen (MEFA) co-administrated with dmLT elicited strong immune responses to each representative adhesin when administered by either the intraperitoneal (IP) (Duan et al., 2017) or subcutaneous (SC) route (Ruan et al., 2015; Duan and Zhang, 2017). Moreover, induced antibodies inhibited adherence with these adhesins. We also observed that when as little as 0.1 μg of the dmLT adjuvant was used, elevated antibody responses were still elicited (unpublished data).

To avoid toxicity, the LTB subunit has been explored for its adjuvanticity when co-administered or fused with antigens. It has reported that LTB subunit expressed in a live attenuated *Salmonella enterica* Typhimurium strain or transgenic tobacco can used as an mucosal adjuvant to induce mucosal and systemic immune response, and increased the efficacy of the *Salmonella*-delivered APEC (Chaudhari and Lee, 2013) or transgenic tobacco derived HPV16-L1 vaccine (Hongli et al., 2013). In addition, intranasally vaccinated the enterovirus 71 (EV71) VP1 subunit (EVP1) plus LTB as adjuvant can significantly enhance EVP1 specific systemic and mucosal antibodies. However, intratracheal instillation (IT) of LTB subunit and purified recombinant Ag85A of *Mycobacterium* tuberculosis induces a Th2-biased immune response, which was generally associated with a poor protection against intracellular mycobacteria when compared to other powerful mucosal adjuvants of the monophosphoryl lipid A of *Salmonella* minnesota (MPLA) and CpG ODN (Todoroff et al., 2013). These results indicated LTB subunit was not potential adjuvant for pulmonary vaccination. The latter vaccine included a genetic fusion of a target antigen gene with the LTB gene; this was performed by substituting the LTA fragment of the native LT genes with nucleotide fragments coding target antigens to generate a chimeric molecule with a LT-like structure. Using this strategy, the GM1-binding LTB pentamer serves as an adjuvant and also as an antigen carrier to target mucosal immunity. It has been reported when intramuscular (IM) the rabies virus-like particles (VLPs)-LTB fusion protein, rabies virus-specific humoral and cellular immune responses were significantly improved in both mouse and dog models (Qi et al., 2015). Similarly, intranasal and IM with the recombinant chimeric protein containing *Mycoplasma hyopneumoniae* three antigens (R1, P42, and NrdF) fused to LTB subunit significantly increase a specific immune response in mice and pigs (Marchioro et al., 2014). These results indicated LTB is a potent mucosal and parental adjuvant when co-administrated or fused with a range of antigens.

**Figure 3:**
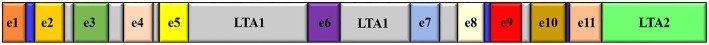
The mapping of epitopes of LTA1 segment. LTA subunit continuous B-cell epitopes were *in silico* identified by using web-based B-cell epitope software as previously described (Ruan et al., 2015). In total, 11 epitopes were identified, the amino acid sequence and position of each epitope is: e1 (1–11): NGDKLYRADSR, e2 (9–21): DSRPPDEIKRSGG, e3 (25–36): RGHNEYFDRGTQ, e4 (42–51): YDHARGTQTG, e5 (54–63): RYDDG YVSTS, e6 (105–115): SPHPYEQEVSA, e7 (140–149): HRNREYRDRY, e8 (156–166): APAEDGYRLAG, e9 (165–177): AGFPPDHQAWREE, e10 (181–193): HHAPQGCGNSSRT, e11 (193–204): TITGDTCNEETQ. The LTA1 segment is gray, LTA2 is green, and the overlap sequence between e1 and e2, e8 and e9, e9 and e10 is blue.

LT enterotoxicity may preclude clinical implementation of native LT as an adjuvant (or an antigen). However, understanding LT adjuvanticity and associated mechanisms that underpin antigen immuno-regulation can lead to the development of a new generation of nontoxic and effective adjuvants. It is believed that both LT ganglioside-binding and toxic enzyme activities are required to upregulate immune responses (Figure 3). LT displays strong adjuvant effects by improving inflammatory cytokine and chemokine secretion and by transiently recruiting immune effector cells to the site of immunization (Ryan et al., 2000; Pizza et al., 2001). LT is also known to negate adjuvanticity by influencing dendritic cell maturation (Petrovska et al., 2003), antigen presentation (Pitcovski et al., 2006), and B- and T-cell activation (Fu et al., 2009). The LTB subunit modulates host immune responses mainly by (i) improving the antigen-presenting capacity of antigen-presenting cells (APC) using major histocompatibility complex class I (MHC-I) (De Haan et al., 2002) and MHC class II presentation pathways (Nashar et al., 2001; Bone et al., 2002), (ii) influencing the maturation and activation of dendritic cells (DCs) and other APCs (Pitcovski et al., 2006), (iii) eliciting strong humoral and cellular responses by activating both B and T lymphocytes, and (iv) stimulating the secretion of cytokines (Yamamoto et al., 2001). It has been reported that the LTB mutant LTB_G33S_ which lacks GM1 receptor-binding activity abolishes any adjuvant ability (Zoeteweij et al., 2006). However, the adjuvant efficacy of mutant LTB_H57S_ which maintains GM1 receptor-binding ability was abolished (Aman et al., 2001). This may suggest that binding to the GM1 receptor is necessary but is not the only mechanism that underlies LTB adjuvant activity. LTA subunit adjuvanticity is predominantly related to its ability to elevate intracellular cAMP. However, the requirement for LTA subunit ADP-ribosylation activity in relation to its adjuvanticity is not well defined. Some catalytically defective and non-toxic LTA mutants still retain strong adjuvant activity (Pizza et al., 1994; Dickinson and Clements, 1995; Douce et al., 1998; Ma, 2016), suggesting that neither induction of cAMP nor enzymatic activity are essential for adjuvant activity. In contrast, Larena et al. (2015) reported that a LT double mutant, which is considered non-toxic induces minimal cAMP production but abrogates its adjuvant effects, indicating that ADP-ribosylation activity is necessary for the adjuvant activities of the LTA subunit. Norton et al. (2012) demonstrated that the LTA subunit alone independent from the A_1_B_5_ holotoxin structure or the presence of the LTB subunit possesses adjuvant activity; whereas de Haan et al. (1998) suggested that the adjuvant activity of non-toxic LTA in combination with the LTB pentamer may be stronger than that of LTA or the LTB subunit alone. Other mechanisms independent of LTA ADP-ribosylation activity or LTB GM1-binding affinity may also exist. The TLR-dependent signaling pathway (Hajishengallis et al., 2005; Hajishengallis and Connell, 2013) along with the Nod2-mediated recognition of the microbiota (Kim et al., 2016) could also be involved in LT adjuvant activity. Additionally, routes of immunization, LT-antigen combinations (whether administered as mixtures, fusion constructs or through chemical coupling) and antigen doses also effect LT adjuvanticity.

## LT as a Carrier or Intracellular Trafficking Vehicle in Advanced Therapies

Within the LT family, the LA1 toxic segment is dispensable for co-assembly of the A2 segment with the LTB subunit, thus making it possible that this segment could be used as a platform to display vaccine antigen epitopes. Indeed, Ruan and Zhang (2013) demonstrated that a triple LT mutant (LTS63K/R192G/L211A), which had LT segments replaced by the porcine ETEC adhesin K88 major FaeG subunit and F18 minor FedF subunit domains, not only formed the A_1_B_5_ holotoxin structure and bound GM1 receptors but also induced antigen-specific protective mucosal immunity against K88+ and F18+ ETEC. Recently, Huang et al. (2018) mapped 11 epitopes of the LTA_1_ domain (Figure 3) and showed that LT with each individual epitope substituted with a foreign epitope exhibited no enterotoxicity but retained GM_1_-binding activity. These results suggest that LT can be potentially used as a novel platform to display heterogeneous epitopes for multivalent vaccines against ETEC and other pathogens.

Biologic therapeutics involves novel medical therapeutic strategies based on the delivery of functional proteins to biochemically relevant sites within diseased cells. Although there has been a rapid increase in clinical biologics, the main challenge pertains to the lack of suitable intracellular trafficking vehicles to deliver active biologic drugs to targeted cells. LT can be used as a novel intracellular trafficking vehicle due to its specific ADP-ribosylation catalytic functionality and cell binding specificity. Liu et al. (2016) demonstrated that the LTA2 segment of the LTA subunit is alone capable of penetrating intestinal and cancer cell membranes. The LTA2 domain together with a fused fluorescent protein can be transported into cells by an A2 domain-mediated transmembrane transporting process similar to a cell-penetrating peptide (CPP). Recently, Lichtenstein and Hocker identified the requisite sequence and associated structures of LTA2 required for the efficient and stable co-assembly of protein cargo with the non-toxic LTB subunit (Lichtenstein and Höcker, 2018). These results suggest that the LT toxin or the LTA2 domain of LTA alone could be used as an intracellular trafficking vehicle for the delivery of biologically active proteins and drugs into cells; in the future, these strategies might be exploited for the treatment of cancer.

## Conclusion

It is noteworthy that recent evidence indicates that LT harbors functions beyond enterotoxic activity. LT also plays a significant role in the enhancement of bacterial adherence, the modulation of immune responses, and the delivery of fused foreign antigens. Recently, LT has been reported to trigger reductions in intestinal epithelial cell viability and to induce apoptosis in a dose- and time-dependent manner in human intestine cell lines (Lu et al., 2017). We expect that additional functions of LT will be unearthed in future studies. While these studies certainly advance our understanding of LT-mediated activities, other questions remain unanswered. For instance, it will be interesting to see whether other pathways, apart from the cAMP signaling pathway, are involved in LT-mediated diarrhea. While LTA and LTB subunits are required to enhance bacterial adherence and adjuvant activities collectively, the precise contribution from individual subunits and detailed mechanisms that underlie these activities remain to be defined. It would appear that binding of the ganglioside GM1 is an essential feature in the facilitation of LT adjuvant properties. It remains to be seen whether other gangliosides (like GD1b, asialo GM1, GM2, and GM3) also participate in the requisite molecular and cellular events that promote adjuvanticity. It will also be interesting to investigate whether Toll-like receptors (TLRs) are involved in LT adjuvant activity due to LT-IIb-B5 pentamer was reported to has ability to activate the TLR2/TLR1 heterodimer and induce NF-κB-dependent production of proinflammatory cytokines (Liang et al., 2007, 2009). Recently, Nod2-mediated recognition of the microbiota was observed to be critical for cholera toxin mucosal adjuvanticity; however, whether LT utilizes the same pathway to elicit mucosal adjuvanticity has not yet been confirmed. Further studies are required to better understand LT bioactivities and to gain a greater insight into the molecular mechanisms pertaining to this important toxin in ETEC pathogenicity.

## Author Contributions

GZ and WZ designed the structure of the review. QD drafted the manuscript. PX prepared the figures. GZ, RN, and WZ critically revised the manuscript.

### Conflict of Interest Statement

The authors declare that the research was conducted in the absence of any commercial or financial relationships that could be construed as a potential conflict of interest.
